# A Comparison of the Effect of Lead (Pb) on the Slow Vacuolar (SV) and Fast Vacuolar (FV) Channels in Red Beet (*Beta vulgaris* L.) Taproot Vacuoles

**DOI:** 10.3390/ijms222312621

**Published:** 2021-11-23

**Authors:** Agnieszka Siemieniuk, Zbigniew Burdach, Waldemar Karcz

**Affiliations:** Institute of Biology, Biotechnology and Environmental Protection, Faculty of Natural Sciences, University of Silesia in Katowice, 28 Jagiellońska St., 40-032 Katowice, Poland; agnieszka.siemieniuk@us.edu.pl (A.S.); zbigniew.burdach@us.edu.pl (Z.B.)

**Keywords:** *Beta vulgaris* L., FV and SV channels, patch-clamp, PbCl_2_, vacuole

## Abstract

Little is known about the effect of lead on the activity of the vacuolar K^+^ channels. Here, the patch-clamp technique was used to compare the impact of lead (PbCl_2_) on the slow-activating (SV) and fast-activating (FV) vacuolar channels. It was revealed that, under symmetrical 100-mM K^+^, the macroscopic currents of the SV channels exhibited a typical slow activation and a strong outward rectification of the steady-state currents, while the macroscopic currents of the FV channels displayed instantaneous currents, which, at the positive potentials, were about three-fold greater compared to the one at the negative potentials. When PbCl_2_ was added to the bath solution at a final concentration of 100 µM, it decreased the macroscopic outward currents of both channels but did not change the inward currents. The single-channel recordings demonstrated that cytosolic lead causes this macroscopic effect by a decrease of the single-channel conductance and decreases the channel open probability. We propose that cytosolic lead reduces the current flowing through the SV and FV channels, which causes a decrease of the K^+^ fluxes from the cytosol to the vacuole. This finding may, at least in part, explain the mechanism by which cytosolic Pb^2+^ reduces the growth of plant cells.

## 1. Introduction

It is well-established that the plant vacuole is a dynamic cellular compartment that can occupy more than 90% of the cell volume and is essential to plant growth and physiological characteristics [1,2,3,4]. Potassium (K^+^) constitutes a chief osmoticum, which plays a key role, among others, in regulating turgor and cell expansion [5]. Vacuolar turgor and vacuolar signalling depend on the concerted actions of the tonoplast transporters and channels. The potassium current across the tonoplast is primarily mediated by two main classes of tonoplast nonselective cation channels (NSCCs): slow-activating (SV) and fast-activating (FV) vacuolar channels. These channels differ in their selectivity, kinetics and activation factors. While the SV channels are permeable to mono and divalent cations; the FV channels are selective solely for monovalent cations [6,7].

Natural processes and human activities are the main causes of environmental contamination by heavy metals. Among the heavy metals, lead is one of the most phytotoxic elements, whose toxic effects have been extensively studied using a variety of plants, particularly with regard to its effects on plant growth and physiological characteristics [8,9,10,11,12]. Although lead is not an essential element for plants, it gets easily absorbed and accumulated in different plant organs [13,14]. One way to reduce heavy metal stress in plants is their detoxication by accumulating and compartmentalising them in the vacuole [15]. The tonoplast includes a number of transporters involved in the translocation of metal ions from the cytosol to the vacuole. The vacuolar pumps and transporters are involved in this process [2].

In spite of the abundant literature on the harmful effects of lead on plant cells, the mechanism of its toxic action has still not been sufficiently explained. Little is known about its effect on the activity of the vacuolar K^+^ channels, which constitute the major conductance of the vacuolar membrane [16] and are involved in plant cell expansion [5].

We began to explore the vacuolar K^+^ channels of red beet taproots due to a suspicion that these channels might be involved in the toxic effect of lead on plant cell growth. It was previously shown that the organic compound of lead (trimethyllead chloride, Met_3_PbCl) modulates the activity of the slow vacuolar (SV) channels in red beet taproot vacuoles [17,18,19]. In the present study, we compared the effect of lead (PbCl_2_) on the slow-activating (SV) and fast-activating (FV) vacuolar channels in red beet (*Beta vulgaris* L.) taproot vacuoles. In contrast to the SV channels, the FV channels have a significant probability of being open in intact resting cells, i.e., at a low cytosolic Ca^2+^ concentration and at voltages close to the resting tonoplast potential [20]. In addition, there are virtually no studies on the effect of lead (Pb) on the activity of the FV channels.

Here, we show that cytosolic lead reduces the currents flowing through the SV and FV channels, which causes a decrease of the K^+^ fluxes from the cytosol to the vacuole. It should also be added that the mechanisms by which cytosolic lead modulates the currents of both channels differ.

## 2. Results

### 2.1. Cytosolic Lead Diminished Whole-Vacuole SV Currents and Single-Channel Activity

**Figure 1:**
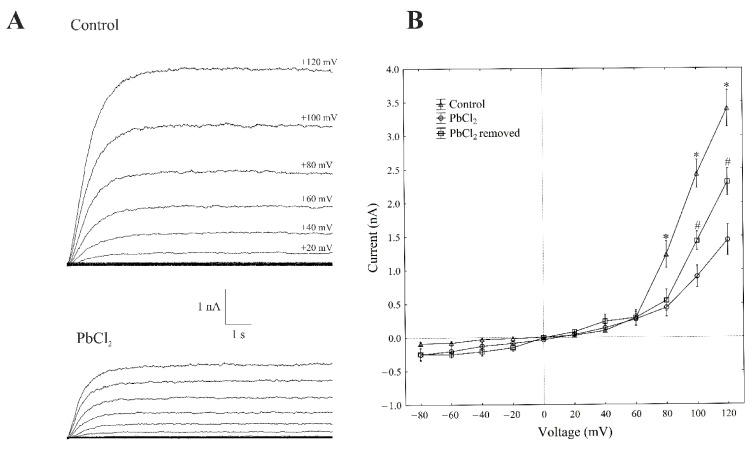
Effect of cytosolic 100-µM PbCl_2_ on the slow-activating currents in red beet taproot vacuoles. (**A**) An example of a whole-vacuole SV current recording in the control bath solution and in the presence of 100-µM PbCl_2_. The whole-vacuole SV current was recorded in a symmetrical 100-mM KCl 1 min after gaining access to the whole vacuole. In order to maintain the same time regime in the variant with PbCl_2_, the control bath solution was changed to a new one with lead within 1 min right after the required patch-clamp configuration was obtained. The macroscopic SV currents were elicited by a series of voltage steps ranging from −80 to +120 mV in 20-mV steps with a holding potential of 0 mV. (**B**) The current–voltage relationships in the absence and presence of 100-µM PbCl_2_ and after PbCl_2_ removal. The means ± SE of at least six different vacuoles are shown. The normal distribution was evaluated using the Shapiro–Wilk test (*p* > 0.05). The statistical differences between the control and PbCl_2_ at the same voltage were analysed using the unpaired *t*-test (*p* < 0.05). An asterisk (*) indicates a significant difference between control and PbCl_2_ variant, whereas a pound mark (#) indicates a significant difference between PbCl_2_ and PbCl_2_ removal variants.

The patch-clamp recordings, which were performed in the whole-vacuole configuration (macroscopic current), showed a typical SV-type channel activity in the vacuoles that had been isolated from *Beta vulgaris* L. taproots. The macroscopic currents exhibited a slow activation (Figure 1A, control) and a strong outward rectification of the steady-state currents at voltages that were more positive than +40 mV (Figure 1B, control).

Here, we decided to use a short period of exposure to lead (within 1 min), because the activity of SV in the channels decreases with time; this phenomenon is commonly known as “rundown” [21,22]. All voltage clamp data concerning the toxic effect of lead was recorded one minute after the control bath solution was replaced with PbCl_2_ solution. A one-minute exposure of the vacuoles to 100-µM PbCl_2_ caused a decrease in the SV outward current in the red beet vacuoles. For example, in the whole-vacuole configuration, the addition of 100-µM PbCl_2_ to the bath solution resulted in a 70% decrease in the SV outward current at +100 mV compared to the control (2.41 ± 0.17 nA, *n* > 5). Interestingly, the removal of PbCl_2_ from the bath medium diminished the inhibitory effect of lead by 60%.

**Figure 2:**
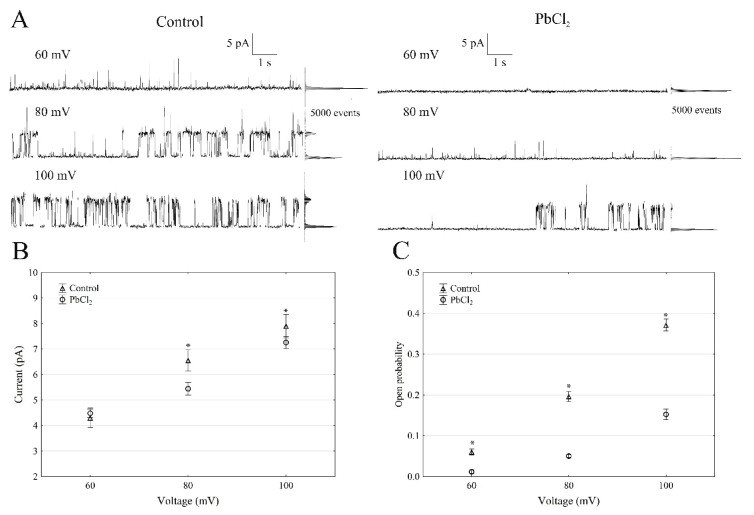
Effect of 100-µM PbCl_2_ on the single SV channel activity in red beet taproot vacuoles. (**A**) The representative single-channel activity of the outside-out patch in symmetrical 100-mM KCl and in the absence and presence of 100-µM PbCl_2_. The current traces are shown at the different membrane voltages (+60, +80 and +100 mV) that are indicated adjacent to the traces. The dotted lines indicate the closing state and the different opening ones. In this patch, at least two channels are opening. The corresponding amplitude histograms are shown to the right of the current traces. (**B**) The current amplitude of a single SV channel current as a function of the voltage (+60, +80 and +100 mV) that was obtained with and without PbCl_2_. The values of the current were obtained as the differences of the maximum of the current histograms and represent the open and closed states of a channel, respectively. The data points are the means (±SE) from at least eight independent experiments. The data did not have a normal distribution (Shapiro–Wilk test, *p* > 0.05); hence, any statistical differences between PbCl_2_ and the control within the same time and voltage were assessed using the nonparametric Mann–Whitney *U* test (*p* < 0.05). An asterisk (*) indicates a significant difference. (**C**) Open probability of the slow vacuolar (SV) channels as a function of the voltage (+60, +80 and +100 mV) in the control bath and in the presence of PbCl_2_. The open probability was calculated as the sum of the channel open times in the current traces, which were normalised to the total time of the traces divided by the number of active channels in a patch using FitMaster software (see, also, Reference [23]). The data points are the means (±SE) from eight independent experiments. The normal distribution was confirmed using the Shapiro–Wilk test (*p* > 0.05). The statistical differences of the open probability values between the control and the PbCl_2_ variants at the same voltage were analysed using the unpaired samples *t*-test (*p* < 0.05). An asterisk (*) indicates a significant difference.

When the single SV channel properties in the cytosolic side-out configuration were analysed (Figure 2), little channel activity could be recorded in the presence of PbCl_2_ (Figure 2A).

The single-channel recordings that were obtained at +100 mV in the control solution and in the presence of PbCl_2_ showed that lead decreased the unitary conductance of single SV channels by 9.8%; in the presence and absence of cytosolic PbCl_2_, the unitary conductance was 71.5 ± 6.1 and 79.2 ± 8.8 pS (*n* > 5), respectively (Figure 2B). However, the open probability of the single SV channels (Figure 2C) decreased by 60% at +100 mV in the presence of PbCl_2_ compared to the control (0.35 ± 0.014). Considering the average number of open SV channels per µm^2^ of the vacuolar membrane, which was calculated as the open probability of a single channel multiplied by the number of channels per µm^2^ (density of the channels; see Material and Methods), it should be stated that PbCl_2_ decreased the mean number of open channels by 87% compared to the control (165 channels per 1000 µm^2^) at +100 mV. Since changes in the macroscopic currents depend on either changes of the single-channel current amplitudes or changes in the mean number of open channels or a combination of both parameters, it should be noted that lead diminished the macroscopic currents of the SV channels via a mechanism that primarily involved a reduced number of open channels, while the unitary conductance of the single channels was decreased to a much lesser extent in the presence of PbCl_2_.

### 2.2. Cytosolic Lead Modulated the Whole-Vacuole FV Currents and Single-Channel Activity

**Figure 3:**
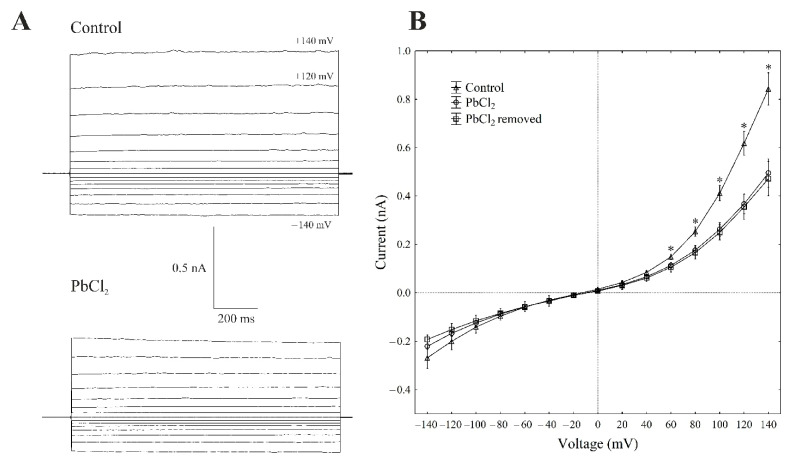
Effect of cytosolic 100-µM PbCl_2_ on the fast-activating currents in red beet taproot vacuoles. (**A**) An example of a whole-vacuole FV current recording in the control bath solution and in the presence of 100-µM PbCl_2_. The whole-vacuole FV current was recorded in a symmetrical 100-mM KCl 1 min after access to the whole vacuole was obtained. In order to maintain the same time regime in the variant with PbCl_2_, the solution was changed to a new one with lead within 1 min after the required patch-clamp configuration was obtained. The macroscopic FV currents were elicited by a series of voltages from −140 to +140 mV in 20-mV steps; a holding potential of −40 mV was applied. (**B**) The current–voltage relationships in the absence and presence of 100-µM PbCl_2_ and after PbCl_2_ removal. The means ± SE of at least eleven different vacuoles are shown. The normal distribution was evaluated using the Shapiro–Wilk test (*p* > 0.05). The statistical differences between the control and PbCl_2_ at the same voltage were analysed using the unpaired *t*-test (*p* < 0.05). An asterisk (*) indicates a significant difference between the control and PbCl_2_ variant.

There is general agreement that, at 100-mM KCl on both sides of the tonoplast and low cytosolic Ca^2+^ (<10 nM), typical whole-vacuole FV currents (Figure 3A) are observed. The whole vacuolar currents that were recorded in these conditions displayed instantaneous currents, which, at the positive potentials (outward current), were about three-fold greater compared to the one at the negative potentials (inward current) (Figure 3A,B).

When PbCl_2_ was added to the bath solution at a final concentration of 100 µM, the FV currents decreased by about 40–60% compared to the control in a range of voltages from +80 to +140 mV. For example, cytosolic lead diminished the outward current through the FV channels by 55% compared to the control (0.40 ± 0.021 nA, *n* > 5) at +100 mV (Figure 3A,B). At the negative potentials, PbCl_2_ did not significantly change the inward currents. Removing PbCl_2_ from the bath medium did not change the inhibitory effect of lead on the FV channels.

**Figure 4:**
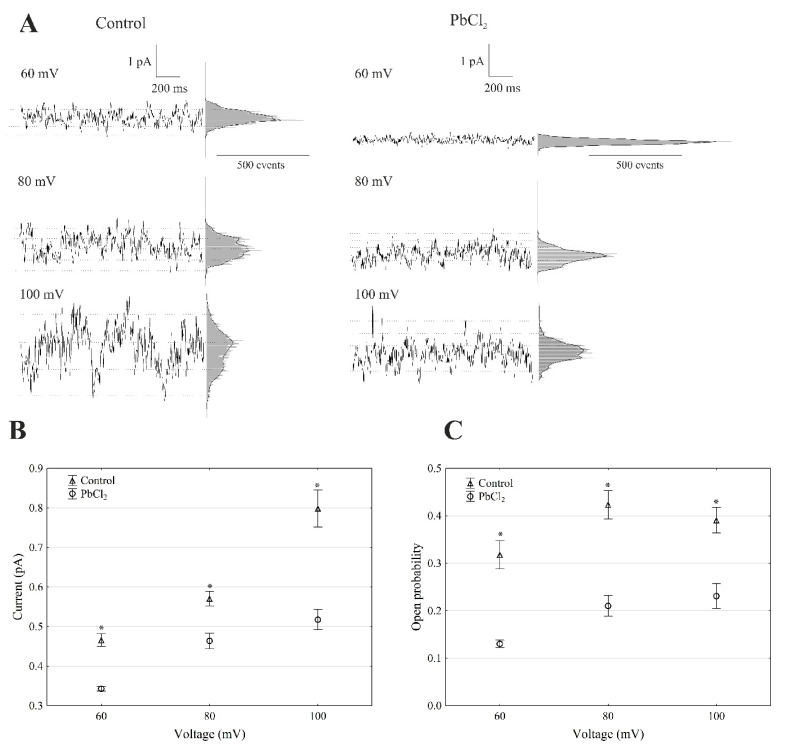
Effect of 100-µM PbCl_2_ on the single FV channel activity in red beet taproot vacuoles. (**A**) The representative single-channel activity of the outside-out patch in symmetrical 100-mM KCl and in the absence and presence of 100-µM PbCl_2_. The current traces are shown at the different membrane voltages (+60, +80 and +100 mV) that are indicated adjacent to the traces. The dotted lines indicate the closing state and different opening ones. In this patch, at least two channels were opening. The corresponding amplitude histograms are shown to the right of the current traces. (**B**) The current amplitude of the single FV channel current as a function of the voltage (+60, +80 and +100 mV) that was obtained with and without PbCl_2_. The values of the current were obtained as the differences of the maximums of the current histograms and represent the open and closed states of a channel, respectively. The data points are the means (±SE) from at least fifteen independent experiments. The data did not have a normal distribution (Shapiro–Wilk test, *p* > 0.05); hence, any statistical differences between PbCl_2_ and the control at the same time and voltage were assessed using the nonparametric Mann–Whitney *U* test (*p* < 0.05). An asterisk (*) indicates a significant difference. (**C**) Open probability of the fast vacuolar (FV) channels as a function of the voltage (+60, +80 and +100 mV) in the control bath and in the presence of PbCl_2_. The open probability was calculated as the sum of the channel open times in the current traces normalised to the total time of the traces divided by the number of active channels in the patch using FitMaster software (see, also, Reference [23]). The data points are the means (±SE) from five independent experiments. The normal distribution was confirmed using the Shapiro–Wilk test (*p* > 0.05). The statistical differences of open probability values between the control and the PbCl_2_ variants at the same voltage were analysed using the unpaired samples *t*-test (*p* < 0.05). An asterisk (*) indicates a significant difference.

The recordings of the single-channel activity, which were obtained with and without lead, showed that PbCl_2_ significantly changed the unitary conductance of the single FV channels (Figure 4A).

The average single-channel conductance in the presence of PbCl_2_ at +100 mV was 35% lower compared to the control (7.98 ± 0.49 pS, *n* > 5) (Figure 4B). The recordings made in the outside-out configuration showed that the open probability of the single FV channels in the variant with lead at +100 mV was 41% lower compared to the control (0.38 ± 0.023, *n* > 5) (Figure 4C). Considering the average number of open FV channels per µm^2^ of the vacuolar membrane, which was calculated in the same way as for the SV channels, it should be stated that PbCl_2_ decreased the mean number of open FV channels by 47% compared to the control (275 channels per 1000 µm^2^) at +100 mV. Taking the above into account, it should be noted that, in the presence of lead, the macroscopic FV currents were diminished via a mechanism that involved the decrease to a similar extent in both the conductance and open probability of a channel.

## 3. Discussion

Lead is known to induce a wide range of toxic effects on plant cells. Many of these effects are due to the fact that, because of its similarity to Ca^2+^ (the similarity of the electron orbitals), Pb^2+^ can interfere with many intracellular processes in which calcium is involved [24].

In the present studies, we have shown that lead blocked the macroscopic current of the SV channels by 70% at +100 mV. In the presence of cytosolic lead, the open probability of the single channels decreased significantly (by 60% at +100 mV) compared to the control, while the unitary conductance of the single channels, which was measured at the same experimental conditions, decreased by only 9.8%. The inhibition of the SV channel currents was also observed in our earlier studies, which concerned the effect of organic lead (Met_3_PbCl) on the SV channels in red beet taproot vacuoles [18,19]. In these studies, we showed that trimethyllead chloride at 100 µM decreased significantly (by about one order of magnitude) the open probability of single channels and only slightly (by ca. 10%) their unitary conductance. From these results, we suggested that the Met_3_PbCl-binding site is located outside the channel selectivity filter and that the inhibitory effect of the compound on SV channels probably results from Met_3_PbCl-induced disorder in compatibility between membrane lipids and membrane proteins.

Taking into account the fact that Ca^2+^ activation of the SV channels is differentially mediated via two cytosolic Ca^2+^-sensing EF-hand motifs, EF-I or EF-II, that are located in the linker between the first and second Shaker-type unit [25,26], it can be speculated that blocking the SV outward currents by cytosolic Pb^2+^ might be due to the conformational changes of the SV channels as a result of the substitution of Pb^2+^ for Ca^2+^ ions in the EF-hand motifs. The question, however, is which one of these two motifs, EF-I or EF-II, is the site where Pb^2^ binds. This dilemma is related to the fact that, so far, studies on the Ca^2+^-binding sites to the EF-hand motif are controversial [6,25,26,27]. In addition, the activity of the voltage-dependent TPC1 (two-pore channel 1) channels can be regulated by both cytosolic and vacuolar Ca^2+^. Cytosolic Ca^2+^ results in the channels opening [28,29], whereas luminal Ca^2+^ prevents their opening [30]. The second possibility, but with significantly less probability, is a case in which cytosolic Pb^2+^ moves through the SV channel (Pb^2+^ has a lower ionic radius than that of K^+^ [31,32,33]) and blocks the channel as luminal Pb^2+^. However, this case is less probable, because the concentration of Pb^2+^ in the bath medium is 1000-fold lower than that of the K^+^ ions.

The FV channels are only permeable for monovalent cations and mediate K^+^ at very low concentrations of cytosolic Ca^2+^ and large voltages of either polarity [16,34,35,36,37]. Moreover, the FV channels are inhibited by the divalent cations from either side of the vacuolar membrane [20,38]. For the FV channels, the results obtained here in the control medium (without cytosolic Pb^2+^) were generally in good agreement with the data that has been published by other authors [20,34,35,39]. However, to the best of our knowledge, the results concerning the impact of cytosolic Pb^2+^ on the activity of the FV channels are now described in the literature for the first time. Our results clearly showed that cytosolic Pb^2+^ blocks the FV channels as a result of a significant decrease in both the open probability (by 41%) and unitary conductance of the single channels (by 35%). Since the structure of the FV channels has not yet been described in the literature, speculating about the impact of cytosolic Pb^2+^ on the activity of these channels is difficult.

It should also be added that, in the case of the SV channels, the inhibitory effect produced by PbCl_2_ could be reversed by 60% at +100 mV after removing the toxic cation, while, for the FV channels, this was not the case (toxic effect of lead was non-reversible). For the SV channels, the partial reversibility of the lead toxic effect may be related to the partial reversibility of Pb^2+^ binding in the sites occupied by Ca^2+^ in the EF-hand motifs (in line with the hypothesis proposed by us above). However, it is difficult to speculate about the non-reversibility of the toxic effect of Pb^2+^ on FV channels, because their molecular structure is not known so far.

Considering the data that was obtained here for both channels, it can be pointed out that cytosolic Pb^2+^ decreased the currents flowing through the SV and FV channels, which caused a decrease of the K^+^ fluxes from the cytosol to the vacuole. This finding may, at least in part, explain the mechanism by which cytosolic Pb^2+^ inhibits the growth of plant cells.

## 4. Materials and Methods

All of the patch-clamp experiments were performed on red beet (*Beta vulgaris* L.) taproot vacuoles that had been isolated using the nonenzymatic method previously described by Reference [40]. The vacuoles were mechanically isolated directly into an electrophysiological chamber (1 mL in volume) by rinsing the surface of fresh tissue slices with the bath solution. The effect of PbCl_2_ on the tonoplast SV and FV channels was studied.

The activity of the SV channels was measured in a bath solution containing 100-mM KCl, 2-mM MgCl_2_, 0.1-mM CaCl_2_, 5-mM MES, 5-mM Tris and 400-mM sorbitol, pH 7.5 and an osmolality of 650 mOsm (control). The solution for filling the pipettes contained 100-mM KCl, 2-mM MgCl_2_, 5-mM MES and 5-mM Tris, pH 5.5, and was adjusted to an osmolality of 580 mOsm with sorbitol. Symmetrical 100-mM K^+^ and micromolar cytosolic Ca^2+^ mediate the outward K^+^ currents (into the vacuole). However, due to the lack of any specific inhibitors of the SV channels, it was almost impossible to distinguish between the K^+^ and Ca^2+^ currents [41]. The concentration of PbCl_2_ (100 μM) was selected as the one that would cause a significant change in the SV activity in the whole-vacuole configuration and, also, because it is below the threshold concentration of many metal salts above which osmotic salt stress can occur [42].

In order to selectively measure the activity of the FV channels, the control bath solution contained 100-mM KCl, 0.5-mM EDTA, 5-mM MES, 5-mM Tris and 400-mM sorbitol, pH 7.5 and an osmolality of 650 mOsm. The pipettes were filled with a solution containing 100-mM KCl, 5-mM MES and 5-mM Tris, pH 6.0, adjusted to an osmolality of 580 mOsm with sorbitol.

Since the FV channels are blocked by higher levels of calcium, CaCl_2_ was excluded from the bath medium, and 0.5-mM EDTA was administered in order to exclusively register the FV current [43,44]. However, unlike those authors, EDTA was not added to the pipette solution, because in the physiological conditions of this experiment, vacuolar Ca^2+^ was present at a high concentration. Moreover, according to Reference [44], an abrupt increase in the inward FV current possibly reflects a sudden decrease in the free vacuolar Ca^2+^ when EGTA becomes the dominant buffer.

The osmolality of all of the solutions was controlled using a cryoscopic osmometer (Semi-Micro Osmometer K-7400, Knauer, Germany).

The experiments were performed in two patch-clamp configurations: a whole-vacuole and excised cytosolic side-out patch using an EPC-7 Plus amplifier (List-Medical-Electronic, Darmstadt, Germany) [21,22]. The current and voltage were in accordance with Reference [45], i.e., any positive (outward) currents indicated an efflux of cations into the vacuole. The reference AgCl electrode was connected to the bath medium via a 3% agar bridge filled with 100-mM KCl. The experimental data were stored using Patch-Master software (HEKA Electronic, Lambrecht, Germany). A five-pole Bessel filter with a sampling frequency of 1–100 kHz was used for signal filtration. The Bessel filter was an integral part of the EPC-7 Plus amplifier. The recordings were sampled at 10 kHz and filtered at a cut-off frequency of 1 kHz. The patch pipettes were prepared from borosilicate glass tubes (Kimax-51, Kimble Products, Toledo, OH, USA) using a pipette puller (model L/M-3-PA, List Medical, Darmstadt, Germany), fire-polished with a CPZ 101 microforge (List Medical, Darmstadt, Germany) and coated with Sylgard (Dow Corning, Midland, MI, USA). The patch electrode resistance was 2–4 MΩ, and the gigaseal resistance was within a range of 5–20 GΩ. Access to the vacuole interior was gained by breaking the tonoplast under the pipette with a voltage pulse within a range of 500–900 mV.

The exchange of the bath solution from the control medium for a new one containing PbCl_2_ was accomplished through the continuous perfusion of the measuring chamber using an SP200 infusion pump (World Precision Instruments, Sarasota, FL, USA). All of the experiments were conducted at room temperature (22 ± 1 °C).

The macroscopic SV currents were elicited by a series of voltage steps ranging from −80 to +120 mV in 20-mV steps with a holding potential of 0 mV. In the case of the whole-vacuole FV currents, a series of voltage from –140 to +140 mV (in 20-mV steps; holding potential of −40 mV) was applied.

Microscopic current traces were used to determine the distribution of the open–close times of a channel and the current amplitudes of the opened channels. The open probability was calculated as the total opening time normalised to the total recording time and the number of active channels in a patch [23,46]. The average number of open channels per µm^2^ of the vacuolar membrane was calculated as the open probability of a single channel multiplied by the number of channels per µm^2^ of the vacuolar membrane (density of the channels). The latter parameter (density) was calculated as the whole-vacuole current divided by the current of the single channel and surface area of the vacuole (in the range of 15–20 µm).

FitMaster (HEKA Electronic, Lambrecht, Germany) and Statistica for Windows (TIBCO Software Inc., Palo Alto, CA, USA, (2017) Statistica (data analysis software system) version 13. http://statistica.io) were used to elaborate and analyse the data.

The free Ca^2+^ concentration of the bath solution was calculated using the Ca-EGTA Calculator v1.2 program (University of California, Davis, CA, USA). All of the details about the composition of the solutions are given in the figure legends.

### Statistical Analysis

The normal distribution was evaluated using the Shapiro–Wilk test (*p* > 0.05). If the normal distribution was confirmed, the statistical differences were analysed using the independent samples *t*-test (*p* < 0.05) or the paired *t*-test (*p* < 0.05). The nonparametric Mann–Whitney *U* test (asymptotic significance two-tailed) was used if the data did not have a normal distribution.

## 5. Conclusions

Using the patch-clamp technique, we compared the activity of two types of tonoplast cation channels, namely the slow-activating vacuolar (SV) and fast-activating (FV) vacuolar channels, in response to PbCl_2_. The patch-clamp experiments were conducted in the whole-vacuole and single-channel configurations in a symmetrical 100-mM KCl on both sides of the tonoplast.

From the experiments described here, we propose that cytosolic lead decreases the current flowing through the SV and FV channels. The current through the SV channels was primarily modulated by a decrease in the SV channels’ open probability, while the current through FV channels was modulated by a decrease in both the unitary conductance of single channels and their open probability. Taking into account the data that was obtained here for both channels, it can be pointed out that cytosolic Pb^2+^ blocks the current flowing through the SV and FV channels, which means a decrease in the K^+^ fluxes from the cytosol to the vacuole. This finding may, at least in part, explain the mechanism by which cytosolic Pb^2+^ inhibits the growth of plant cells.

## Data Availability

Not applicable.
